# Supplementary figures and images for: Ambroxol Treatment Suppresses the Proliferation of Chlamydia pneumoniae in Murine Lungs
Source: Microorganisms. 2021 Apr 20;9(4):880. doi: 10.3390/microorganisms9040880 (PMC8074272; doi:10.3390/microorganisms9040880)

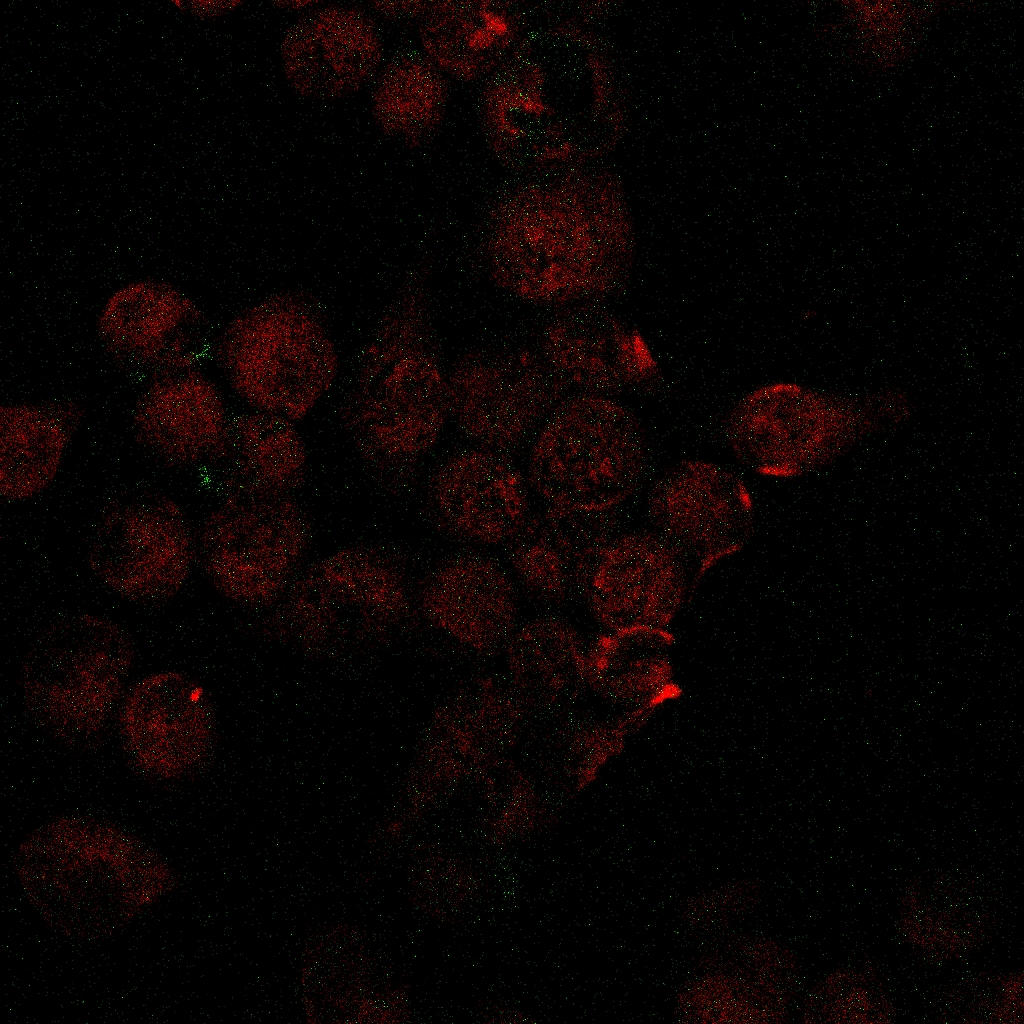

Supplement: Supplementary file 1 [file microorganisms-09-00880-s001.zip › SP-A.jpeg]

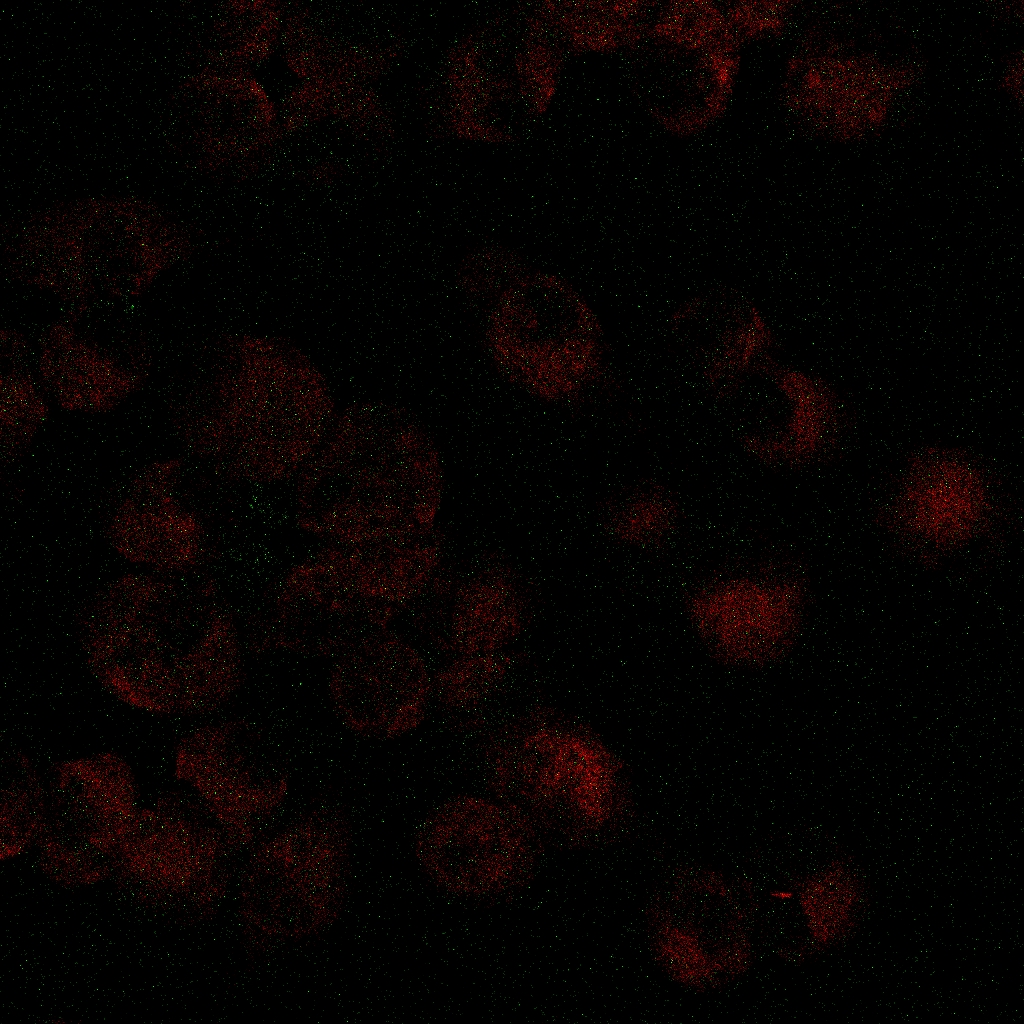

Supplement: Supplementary file 1 [file microorganisms-09-00880-s001.zip › SP-D.jpeg]

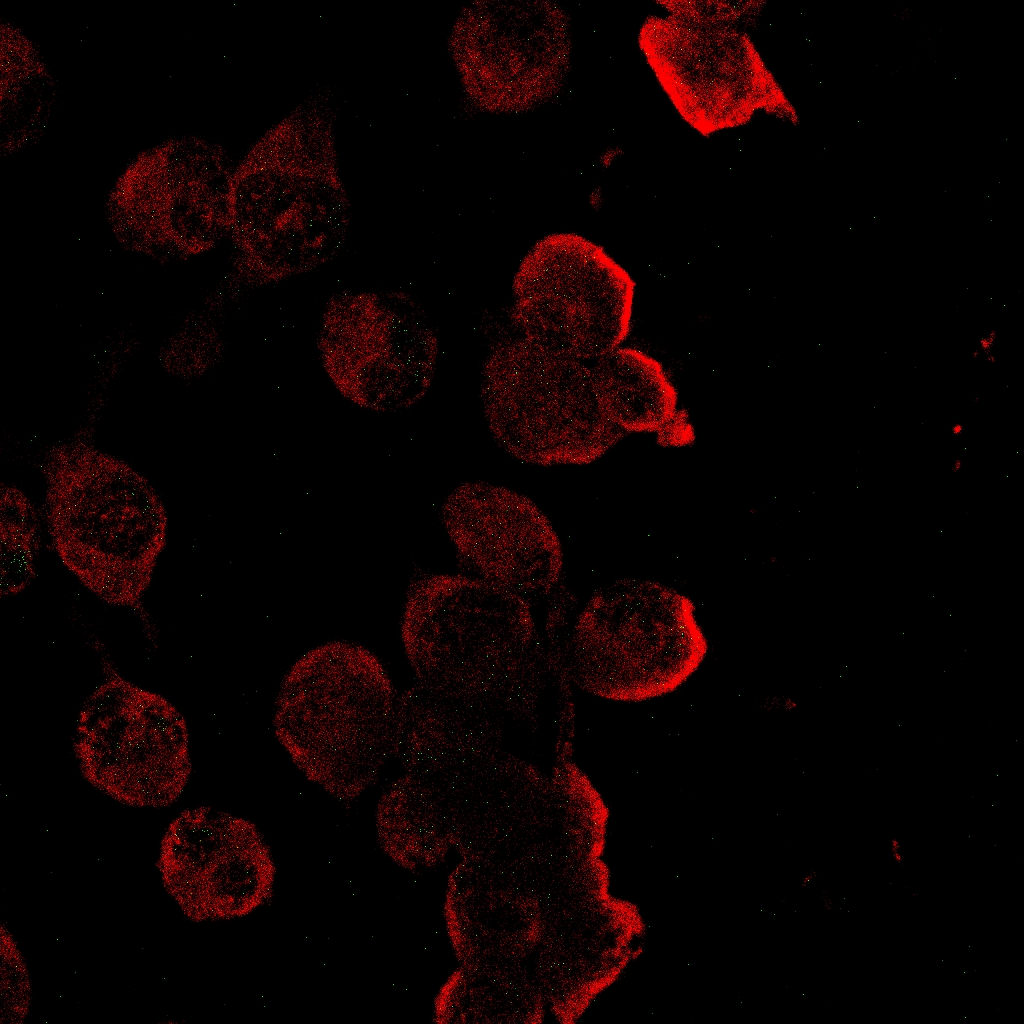

Supplement: Supplementary file 1 [file microorganisms-09-00880-s001.zip › untreated.jpeg]
